# An Instance Segmentation-Based Method to Obtain the Leaf Age and Plant Centre of Weeds in Complex Field Environments

**DOI:** 10.3390/s21103389

**Published:** 2021-05-13

**Authors:** Longzhe Quan, Bing Wu, Shouren Mao, Chunjie Yang, Hengda Li

**Affiliations:** 1 College of Engineering, Northeast Agricultural University, Harbin 150030, China; 13204661592@163.com (B.W.); mao_shouren@163.com (S.M.); yj1149037864@163.com (C.Y.); lihengdade@163.com (H.L.); 2 College of Engineering, Anhui Agricultural University, Hefei 230036, China

**Keywords:** weeds, phenotype, deep learning, image segmentation

## Abstract

Leaf age and plant centre are important phenotypic information of weeds, and accurate identification of them plays an important role in understanding the morphological structure of weeds, guiding precise targeted spraying and reducing the use of herbicides. In this work, a weed segmentation method based on BlendMask is proposed to obtain the phenotypic information of weeds under complex field conditions. This study collected images from different angles (front, side, and top views) of three kinds of weeds (*Solanum nigrum*, barnyard grass (*Echinochloa crus-galli*), and *Abutilon theophrasti* Medicus) in a maize field. Two datasets (with and without data enhancement) and two backbone networks (ResNet50 and ResNet101) were replaced to improve model performance. Finally, seven evaluation indicators are used to evaluate the segmentation results of the model under different angles. The results indicated that data enhancement and ResNet101 as the backbone network could enhance the model performance. The *F*_1_ value of the plant centre is 0.9330, and the recognition accuracy of leaf age can reach 0.957. The *mIOU* value of the top view is 0.642. Therefore, deep learning methods can effectively identify weed leaf age and plant centre, which is of great significance for variable spraying.

## 1. Introduction

Weeds are regarded as one of the biggest threats to crop growth because they compete with crops for nutrients, light, water and other resources [1,2]. At present, the control of weeds is mainly based on the traditional uniform spraying of herbicides. This method has caused excessive use of herbicides and environmental pollution. According to the principle of herbicide and plant physiology [3], the phenotypic information of weeds is closely related to the herbicide dosage. The age of weeds affects the optimal dosage of herbicides to be used [4]. Although the upper limit of herbicide application can eliminate weeds of different ages, this scenario may involve excessive use of herbicides. Moreover, the plant centre is the area formed by the overlapping of the petiole and stem of the top leaves of the weed. This area is mostly new tissue, with a large number of stomata, with a thin waxy layer, which is more sensitive to herbicides has a higher capacity for herbicide absorption, and is the best location for herbicide delivery, especially for weeds above four leaf age [5]. Therefore, accurate identification of weed leaf age and plant centre is of great significance to reduce the use of herbicides, improve the utilization rate of herbicides, and avoid environmental pollution.

Automatic identification and classification of weeds are the basis for automatic weed removal. In recent years, machine vision [6] has been widely used in the agricultural field. Researchers have developed different algorithms for the identification and location of weeds [7,8]. For traditional computer vision algorithms, researchers mostly identify weeds based on single features such as colour features, texture features and multispectral features [9]. However, due to the similarity between weeds and crops, the accuracy of using a single feature to detect weeds is not high, and it is not suitable for random targets and variable field environments [10]. Deep learning methods can extract multiscale and multidimensional spatial feature information of weeds through convolutional neural networks, which solves the disadvantages of traditional methods in feature extraction and is widely used in weed identification. To improve the accuracy of weed identification and classification, researchers have carried out much research on algorithm improvement, data collection and dataset construction. Chen et al. [11] proposed a method combining multifeature fusion and a support vector machine to identify and detect corn seedlings and weeds to improve the accuracy of the model from the perspective of algorithm improvement. Quan et al. [12] collected full-cycle, multi-weather and multi-angle data sets to improve the accuracy of the Faster R-CNN model for detecting weeds. Dian et al. [13] conducted research from the perspective of data construction, used deep learning with unsupervised data labelling to detect weeds in unmanned aerial vehicle (UAV) images obtained from bean fields and spinach fields, and obtained results close to the labelling of traditional supervised training data. Deep learning technology has advantages over traditional computer vision algorithms in weed recognition and localization. These methods can only obtain the specific location and cannot obtain the morphological structure information of weeds. However, related research results show that the morphological structure of weeds and other details are important for further improving weeding performance and saving herbicide dosage.

Plant phenotyping is an emerging science that links genomics with plant ecophysiology and agronomy, and plays an important role in genetics, botany and agronomy. The study of plant phenotypes, mainly divided into plant organ phenotypes and whole plant phenotypes, has been widely used in various fields. Among them, the morphology, growth and counting of plants are an important part of the phenotype of the above-ground organs of plants, and play an important role in understanding the morphological structure of plants. Researchers often use the leaf area and leaf shape of a plant to estimate information such as the plant’s growth status and yield [14]. In recent years, the application of computer vision technology to analyse plant phenotypes has become a research hotspot in this field. Leaf count has always been an important challenge for plant phenotype research. Bell et al. [15] segmented leaves through edge classification and counted the leaves, which also achieved satisfactory results for plant overlap. Dobrescu et al. [16] proposed a multitask deep learning framework for plant phenotypes and achieved promising results for leaf counting. To further improve the performance of the model and reduce the labour cost of annotated data, Zhang et al. [17] used 3D synthetic plant images to improve the performance of rosette leaf count and reduce the average absolute error. The above research has achieved good results in leaf segmentation counting. However, the plant images in these studies were collected under indoor conditions, and such images usually exhibit a pure background and light uniformity. While the farmland environment is complex, the differences between plants and the mutual occlusion between leaves will affect our imaging quality and model performance, so it is still a great challenge to obtain weed leaf age in a complex field environment.

Deep learning is an emerging field of machine learning aimed at solving image data analysis problems. The instance segmentation algorithm based on deep learning can classify each object pixel by pixel, and the output is the mask and bounding box of the target object [18], which is particularly suitable for solving the problem of leaf adhesion and occlusion. Therefore, instance segmentation is a common method to obtain farmland phenotypic information in the field of deep learning. Field phenotypic researchers have mainly studied economic crops such as grapes, apples, flowers, strawberries. Researchers used deep learning algorithms to segment and count different varieties of grapes, which could adapt to the complex environment of farmland and avoid the problem of multiple counting [19,20]. Gené-Mola et al. [21] used instance segmentation network and dynamic structural photogrammetry technology to segment and locate apple images in the field. The results of fruit location in 3D point cloud showed that *F*_1_ score was 0.88, which effectively reduced false positives in the process of recognition. Mask R-CNN, an instance segmentation algorithm proposed by He et al. [22], is a typical representative of a two-stage segmentation network. The researchers used the improved Mask R-CNN model to obtain the phenotype of apple flowers and strawberries and achieved good segmentation under occlusion and overlap conditions [23,24]. The BlendMask model proposed by Chen et al. exhibited a higher segmentation performance on the COCO dataset [25] than the Mask R-CNN [26]. BlendMask combines the ideas of top-down and bottom-up methods. Moreover, BlendMask employs fully convolutional one-stage object detection (FCOS) [27], which eliminates the calculation of the position-sensitive feature map and mask feature. Thus, the inference time does not increase with the number of predictions, as in the traditional two-stage method. The above studies considered the complex environment of farmland and proposed many good methods to obtain phenotypic information, while the morphological structure characteristics of crops segmented by these studies were relatively uniform. However, weeds are polymorphic, weeds of different varieties and leaf ages are quite different, and their growth positions are randomly changeable. Therefore, weed segmentation at different scales in complex field environments is relatively rare. In this study, a new instance segmentation model was used to segment weeds under a complex field environment to obtain weed species, leaf age and plant centre.

In view of the above research, our goal is to propose a weed phenotype acquisition method based on an instance segmentation model that can provide weed species, leaf age, and plant centres in a complex field environment. More importantly, this study considered the effects of different data sets, different instance segmentation models and model optimization, different backbone networks, different data shooting angles and different weed morphological structures on the model performance.

(1) Construct weed datasets with different shooting angles and different growth stages in a complex field environment.
(2) Compare different instance segmentation models, select the optimal model and optimize parameters.
(3) To explore whether data enhancement and the use of ResNet101 combined with FPN architecture for feature extraction can enhance the model performance.
(4) To explore the influence of data collection from different angles (front view, side view, top view) and morphological characteristics of weeds on weed phenotypic segmentation under a complex field environment were discussed.

## 2. Materials and Methods

### 2.1. Image Acquisition

**Figure 1:**
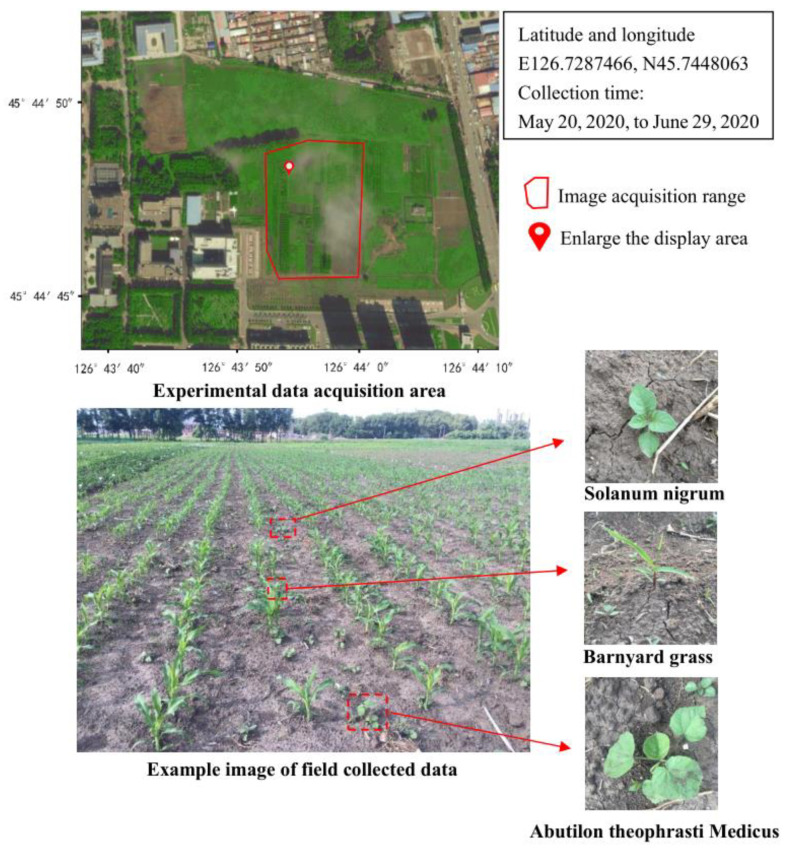
Image collection area and collection varieties.

Field data were collected from 20 May 2020, to 29 June 2020, at the two-leaf stage after crop planting in Xiangfang District (E126.7287466, N45.7448063). Xiangfang District is located in the northeast plain and is the major growing region for economic crops such as maize and soybean. The data collection work was selected from the more common maize fields of Dongnong 257 in Northeast China, where the area is monopoly crop, mechanically sown, with a maize plant gap of 20–30 cm, a monopoly spacing of 60–65 cm and a plant height of 6–13 cm. The following three kinds of weeds were selected in this study: *Solanum nigrum*, barnyard grass (*Echinochloa crus-galli*), and *Abutilon theophrasti* Medicus. *Solanum nigrum* is an annual dicotyledon, barnyard grass (*Echinochloa crus-galli*) is an annual herb, and *Abutilon theophrasti* Medicus is an annual subshrub weed. The three kinds of weeds are commonly found in the farmland of Xiangfang District, as shown in Figure 1. Weeds in the field are mostly two- five leaf age, so we only randomly collected weed data before the five-leaf stage.

**Figure 2:**
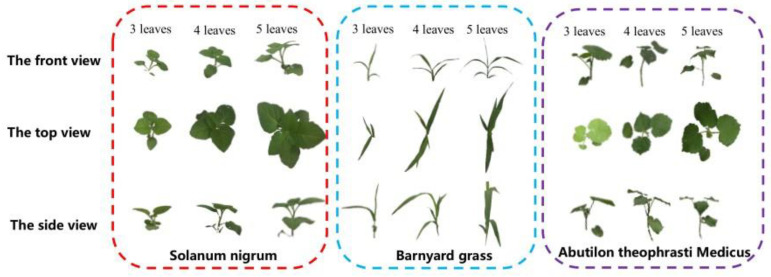
Front, top, and side views of the three weeds.

When collecting the dataset, the shooting angle in the field [28] and growth stage of the weeds may affect the dataset accuracy [29]. The ideal viewing angle for image acquisition is 57°, but the position and shape of weeds in the field are complex and changeable, and the shape of the same object is different under different shooting angles, as shown in Figure 2, so it is necessary to know what effect different orthogonal angles have on the accuracy of the dataset. Moreover, we collected data from three angles, corresponding to the front, side and top views [28], which could clarify the comprehensive information of weeds and enable the model to cope with the requirements of operations from different angles. In this paper, the direction parallel to the monopoly is specified as the direction of the front view, and the direction perpendicular to the monopoly is specified as the direction of the side view, and the angle between the main view and the and side view is 90°, which is measured with a measuring angle plate during data collection. Table 1 shows the collection of weed images with different leaf ages under different weather conditions, different angles and different growth stages every 2 to 5 days during the collection period. The camera of the iPhone 6s Plus device, with a focal length, maximum aperture, and maximum resolution of 4.2 mm, f/2.2 and 4032 × 3024 pixels, respectively, was used to capture images, and the weed images were stored in the JPEG file format. JPEG compression was adopted for the images because it is a widely accepted method to reduce file size with a selectable loss of quality [24]. The use of mobile phones as image acquisition equipment for the identification of relevant feature areas is also common in the industry and this method of image acquisition is convenient and quick [30,31]. iPhone 6s Plus has relatively mature technology, and its anti-distortion ability is relatively high, which perfectly suited to our operational needs. When collecting data, we marked the sample variety, leaf age, collection time, collection angle, collection weather, and temperature into the sample data.

### 2.2. Dataset Construction and Annotation

When training the network, the images need to be screened and adjusted to a uniform size to meet the training requirements of DCNN [32]. First, discard some inappropriate annotated images. Second, to avoid changing the morphology of plants when adjusting the size of plants, the specific steps were as follows: (1) the original image size collected in the field was 4032 × 3024; (2) the image was cut to 3024 × 3024; and (3) the size of the image was adjusted to 1024 × 1024. Third, we should keep some blurred, occluded and incomplete images as negative samples when building a dataset. Finally, 5700 images were selected from 5856 images. Since the model needs to be tested, 600 images are retained for model evaluation, and 900 images are used to verify the accuracy of the model for leaf age identification.

**Figure 3:**
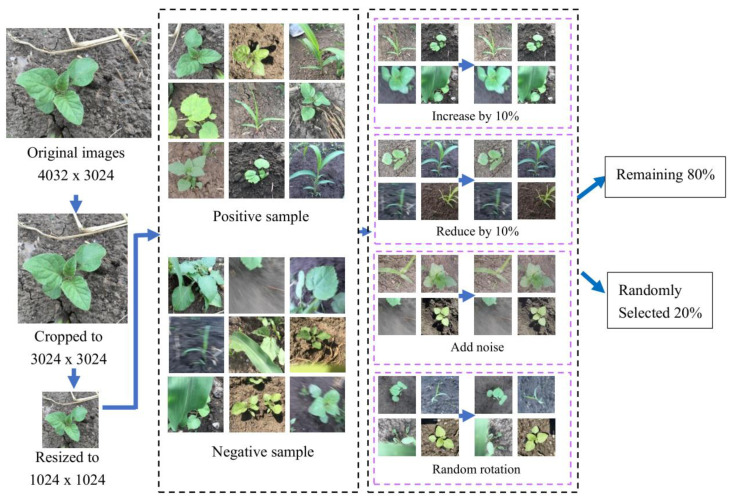
Data enhancement. Note: The size of the original images is 4032 × 3024. After cropping the images to a size of 3024 × 3024, the images are resized to 1024 × 1024. The collected images include positive and negative samples. The acquired images are brightened and darkened by 10% and subjected to increased noise and random rotation. Finally, the dataset is randomly divided into training and verification sets at a ratio of 8:2.

Data enhancement can further enrich the sample image, make the dataset more representative, and more accurately reflect the real situation of the field data [12], therefore, this study used the method of data enhancement to expand the dataset, improve the training precision of the model and reduce overfitting [33]. The specific operation is shown in Figure 3: randomly flip the image, add noise, adjust the brightness to brighten 10%, darken 10%. The structure and proportion of the original dataset remained unchanged when data enhancement was implemented, and 6000 data-enhanced pictures were obtained. Therefore, two datasets were prepared, with and without data enhancement. Both datasets were randomly divided into training and verification sets at a ratio of 8:2 [24,30].

**Figure 4:**
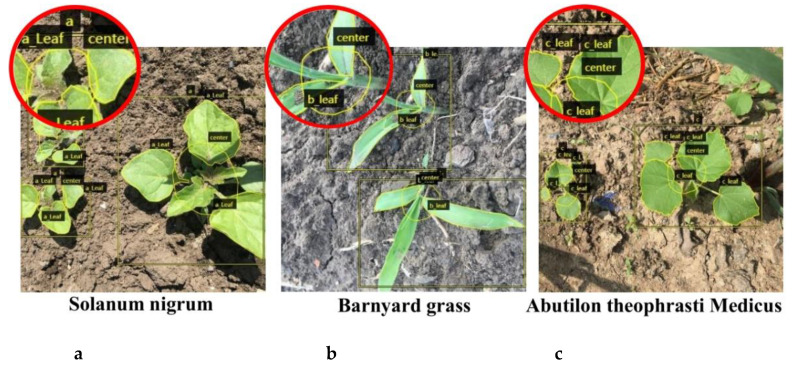
Sample of image annotation in the segmentation process. Note: Leaf of *Solanum nigrum* (a_leaf), barnyard grass (b_leaf), and *Abutilon theophrasti* Medicus (c_leaf). Plant centre (centre). *Solanum nigrum* (**a**), barnyard grass (**b**), and *Abutilon theophrasti* Medicus (**c**).

The VGG Image Annotator labelling tool [34] was used for the annotation, as shown in Figure 4. The plant centre was surrounded by irregular polygons that can be more in line with crop morphological structure characteristics. The number of weeds in an image is uncertain under actual working conditions, and the image may contain multiple weeds. Therefore, the number of masked leaves in the picture could not be used to calculate the leaf age of a single weed. In this study, we surrounded the leaves of weeds with irregular polygons, marked the outline of the outermost layer of a single weed with rectangular frames, and did not mask the rectangular frames. The number of leaf masks in the rectangular frames was calculated, which was the leaf age of the weed. The labels were divided into seven categories (Figure 4).

### 2.3. Weed Instance Segmentation Model

**Figure 5:**
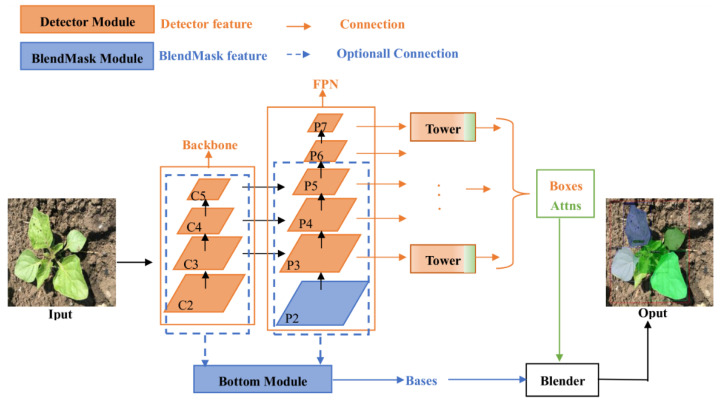
Model structure of BlendMask.

Instance segmentation algorithms are one of the most challenging tasks in computer vision because they not only have to perform classification at the pixel level for semantic segmentation but also have some target detection characteristics. Common instance segmentation models are divided into two categories: one-stage instance segmentation models and two-stage instance segmentation models. The representative of the two-stage instance segmentation model is Mask RCNN. The one-stage instance segmentation model mainly includes YOLACT, PoParMask, CenteMask, Solo, BlendMask and so on. The BlendMask model is a one-stage dense instance segmentation algorithm that combines instance-level information with lower-level fine-granularity semantic information. BlendMask is composed of a one-stage target detection network FCOS [27] and a mask branch. Figure 5 shows the model structure of BlendMask. The mask branch has three parts: The bottom module is used to process the bottom features to generate the score maps, the top layer is attached to the box head of the detector to generate the top level attention corresponding to the base, and the blender module is used to fuse the base and attention. BlendMask combines top-level and bottom-level information. The top level corresponds to a broader receptive field, such as the posture of the whole weed plant. Because the top level is a rough prediction, the resolution of the top-level ROI is relatively small. Compared to Mask RCNN fixed output of 28×28, BlendMask output resolution can be higher because its backbone is not limited by FPN. The bottom level corresponds to more detailed information, such as the position and centre of the weed, which can retain better position information. BlendMask combines the concepts of the top-down and bottom-up methodologies, thereby combining rich instance-level information with accurate dense pixel features [26].

BlendMask can establish deep neural network models of different depths by implementing different weight layers. The deep learning network models applied at present include AlexNet, ZF, GoogLeNet, VGG, and ResNet. Although the deeper the number of network layers, the higher the accuracy, the deeper the number of network layers, and the training and detection speed of the model will decrease. The residual network does not increase the number of model parameters, the problem of training degradation can be alleviated, and the model convergence can be accelerated [24]. Therefore, in this study, ResNet50 and ResNet101 combined with the FPN were used as the backbone networks to extract the features of the weed images.

### 2.4. BlendMask Training Model

Transfer learning can use previous knowledge to solve new but similar problems much more quickly and effectively [35]. It is an effective way to reduce training labour and cost and improve training efficiency. Therefore, before training BlendMask, we introduced a pretraining model based on the COCO dataset [25] through transfer learning. The COCO dataset has 328,000 images, including 91 categories. The pretraining model extracted the weights after training on the COCO dataset, based on which the established datasets were retrained. Transfer learning can adjust the model parameters to a better state. The programming language used in the BlendMask model of this experiment is python 3.6, and the deep learning framework used is pytorch 1.7. The deep learning algorithm uses the pycharm community version 2020 platform. The BlendMask model was implemented using the AdelaiDet open source toolbox based on detectron2. The experiment was performed on the Ubuntu 18.04 operating system over a six-core Intel Core i7-8700K @ 3.70 GHz processor, 32 GB of memory, and a GPU built by NVIDIA GeForce (Santa Clara, CA, USA), along with the NVIDIA GeForce RTX 1080 Ti graphics card. The pretraining network parameters are listed in Table 2.

### 2.5. Training and Evaluation

The momentum and initial learning rate for BlendMask were set as 0.9 and 0.01, respectively, and the training BatchSize was set as 4. After the parameters were set, training was conducted for 12 rounds, with 10,000 iterations being implemented in each round. The basic framework of BlendMask involved either ResNet50 or ResNet101.

The evaluation aimed to examine the ability of the algorithm to identify the weed leaf age and plant centre in the image. For the evaluation, we used seven key indexes: precision rate (*P*) (Equation (1)), recall rate (*R*) (Equation (2)), *F*_1_ (Equation (3)), intersection over the union (*IOU*) (Equation (4)), average precision (*AP*), mean average precision (*mAP*) (Equation (5)), and mean intersection over the union (*mIOU*).

The precision rates (*P*) and recall rates (*R*) can be defined as follows:

(1) $$P=\frac{TP}{TP+FP}\;$$

(2) $$R=\frac{TP}{TP+FN}$$

where “true positive (*TP*)” and “false positive (*FP*)” indicate the number of positive and negative results detected as positive, respectively, and “false negative (*FN*)” indicates the number of positive results detected as negative. The metric function (*F*_1_) of the precision and recall rates can be defined as follows:

(3) $$F_{1}=\frac{2P*R}{P+R}$$

**Figure 6:**
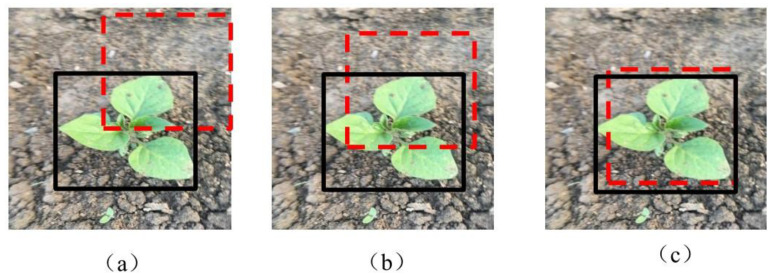
Visual example of intersection over the union. Note: The red dashed box and black solid-line box represent the prediction of the detection result and ground truth, respectively. The overlap between the two boxes is visible, and the different *IOU* values can be determined. (**a**–**c**) represents each of the three different *IOU* scores, (**c**) indicates the optimal case.

To address the problem of multiclass imbalance, we averaged the seven classification indicators [36]. To more extensively evaluate the model algorithm, the intersection over union error (*IOU*) was considered to examine the measurements. In general, the *IOU* measures the overlap between two bounding boxes. Figure 6 illustrates the calculation of the overlap degree between the weed prediction box and real box on the ground. The detection performance of the model is evaluated considering the mean accuracy (*mAP*) [37]. The *mAP* can clearly reflect the performance when it is related to the target position information and category information of the target in the image. The AP can be calculated for each category separately, and the value for each category can be averaged to calculate the *mAP*. Two thresholds are evaluated according to existing technology, the thresholds were set as 0.5 and 0.7 in this study [30,38]. When the *IOU* threshold was equal to or greater than 0.5 and 0.7, the *mAP* was defined as AP50 and AP70, respectively. The *IOU* and *mAP* were defined as follows:

(4) $$IOU=\frac{Area\;Overlap}{Area\;Union}\;\;$$

(5) $$mAP=\frac{1}{N}\cdot \overset{N}{\underset{i=1}{\sum }}AP_{i}$$

where *N* represents the number of images.

## 3. Results and Discussion

**Figure 7:**
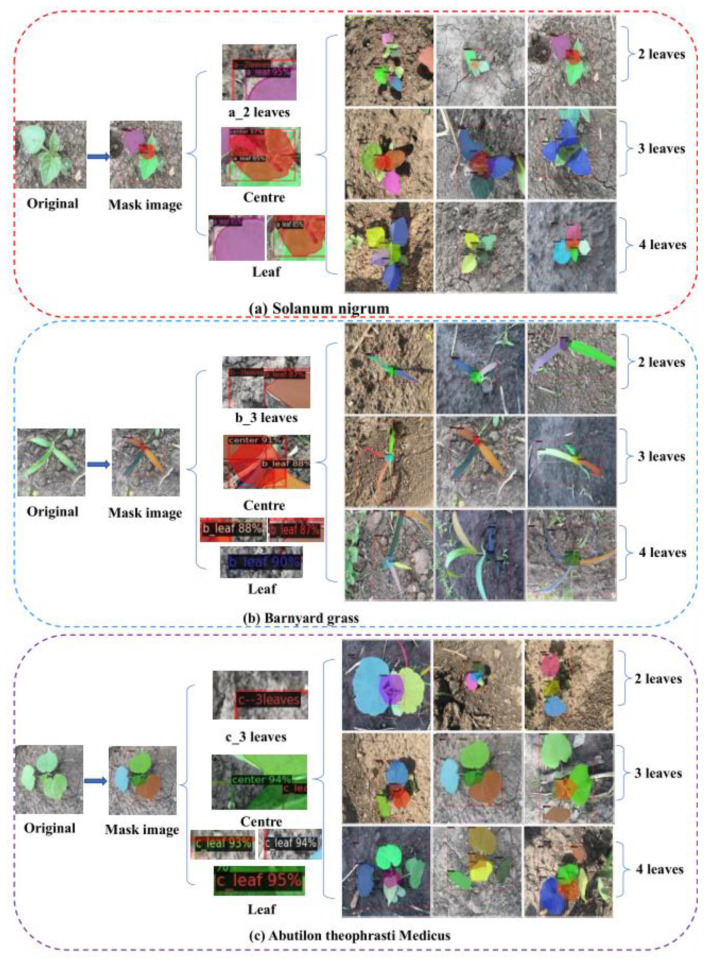
Segmentation results for the three weeds with different leaf ages. Note: The left area of the image shows the leaves, plant centre and leaf age for each type of weed mask. (**a**–**c**) show the recognition results for the different types of weeds with different leaf ages.

In this study, the instance segmentation method was used to obtain the species, leaf age, and plant centre of weeds. The results are shown in Figure 7. The performance of instance segmentation will determine the effect of weed recognition, so the following research is carried out for instance segmentation networks. First, we compare the six typical instance segmentation models. Second, we optimize the hyperparameters of the optimal model to improve network performance. Finally, seven evaluation indexes are selected to evaluate the performance of the optimized model. The detailed research results are shown below.

### 3.1. Comparison of Instance Segmentation Models

**Figure 8:**
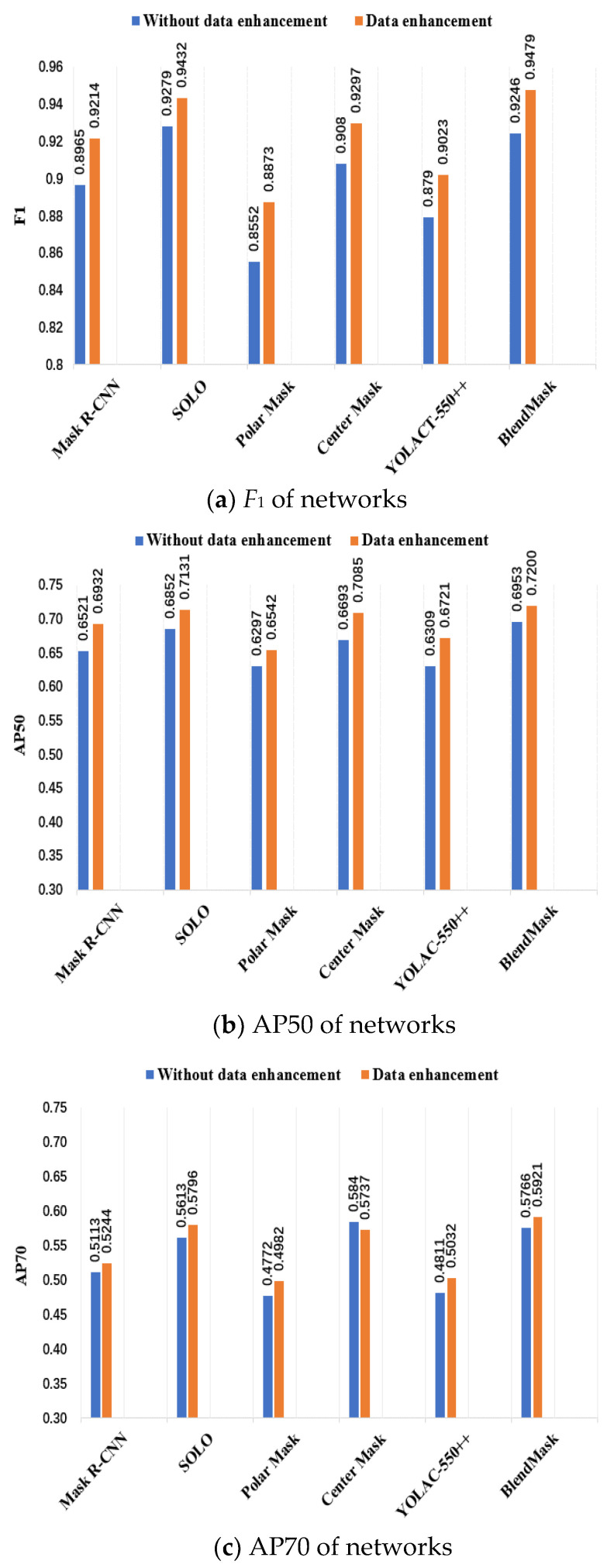
Detection results for different instance segmentation models. Note: The backbone network of the six models (YOLACT, PolarMask, BlendMask, CenterMask, SOLO and Mask R-CNN) is ResNet101. “Without data enhancement” and “Data enhancement” refer to the models trained using 4200 unenhanced datasets and 6000 enhanced datasets, respectively. When the *IOU* threshold is greater than or equal to 0.5 and 0.7, the *mAP* is defined as AP50 and AP70, respectively.

To verify the effectiveness of the proposed method for weed segmentation, six typical instance segmentation algorithms, including Mask R-CNN, SOLO [39], PolarMask [40], CenterMask [41], YOLACT [42] and BlendMask, were compared. This study will identify weed phenotypic information in complex field environments. To further improve the adaptability of the model, the image data are enhanced. The above six algorithms are trained on two datasets: one is the original image, and the other is the enhanced image. To examine the recognition effect of the model in a complex field environment, the images in the test were those without enhancement. Figure 8 shows the *F*_1_, AP50, AP70 values of the model.

**Figure 9:**
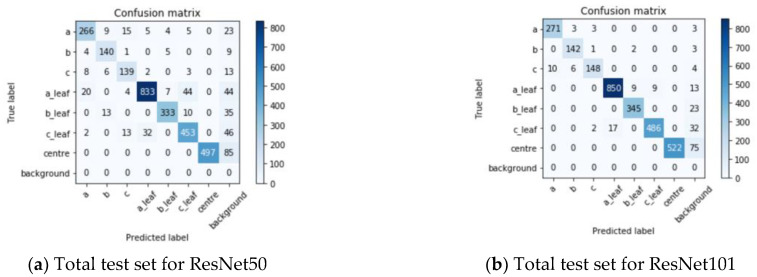

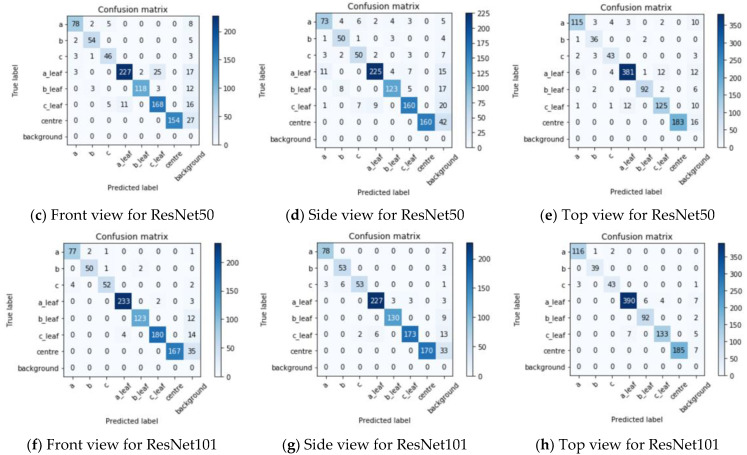
Confusion matrix of the detection results of ResNet50 and ResNet101 in the case of data enhancement. Note: The BlendMask with ResNet50 and ResNet101 frameworks are expressed as ResNet50 and ResNet101, respectively. a_leaf, b_leaf, and c_leaf represent the leaves of *Solanum nigrum*, barnyard grass, and *Abutilon theophrasti* Medicus, respectively. Moreover, a, b, and c represent *Solanum nigrum*, barnyard grass, and *Abutilon theophrasti* Medicus, respectively, and the centre represents the plant centre of each weed.

Figure 8a shows the *F*_1_ values of six instance segmentation networks. From the figure, it can be seen that data enhancement can increase by at most 3.21% and by at least 1.53% compared to that without enhancement. The effect of data enhancement is generally higher than that without enhancement. For the convenience of description, in the following research we will write YOLACT-550++ as YOLACT. In the case of data enhancement, the *F*_1_ values of Mask RCNN, SOLO, CenterMask and BlendMask are greater than 0.92, while the *F*_1_ values of YOLACT and PolarMask are the lowest. To further analyse the recognition performance of multiclassification target location and category information, Figure 9b,c show the AP50 and AP70 of six instance segmentation networks. Through comparison, it can be seen that the value of AP50 is higher than that of AP70. Choosing an *IOU* threshold greater than or equal to 0.5 is more suitable for this study. In the result of Figure 8b, we can see that in the case of data enhancement, the AP50 values of the six models are between 65% and 72%, which can meet the needs of weed case segmentation. The AP50 values of BlendMask, SOLO and CenterMask are greater than 70%.

Field weeds are visual objects with complex structures and rich texture features. Even within the same species, there are great differences in morphology and colour. Data enhancement can improve the generalization ability of the model, reduce overfitting, and improve the adaptability of the model to complex field environments. The main reasons for the good recognition of BlendMask, SOLO and CenterMask are: BlendMask combines the idea of top-down and bottom-up methods to fuse rich information at the instance level and accurate dense pixel features, so it is very suitable for the situation of leaf occlusion in this study. SOLO uses instance categories to realize direct instance segmentation, which is free from the influence of target detection. While, CenterMask is also a one-stage instance segmentation model that contains both global and local image methods of YOLACT and PolarMask. It can complete the instance segmentation of different objects in the case of pixel-level feature alignment, so it has achieved good segmentation results. However, CenterMask is still not completely out of the influence of target detection, so the AP50 value of CenterMask is second only to BlendMask and SOLO.

The main reasons for the poor recognition effect of YOLACT and PolarMask are that YOLACT is a one-stage instance segmentation network that uses a global image-based method to process the image. This method can better retain the location information of the object; however, for the case of leaf occlusion, it may not be able to accurately locate each weed leaf, resulting in the occluded leaves below being recognized as the leaves of the foreground mask, causing errors. PolarMask is also a one-stage instance segmentation model. The contour of the object is described by the polygon composed of rays emitted from the centre of the object. However, the weed is polymorphic, the morphological structure is complex, and the plant centre is also special. This method may not accurately describe the edge of the object. When connecting the endpoints of each ray, some local segmentation information will be lost, which makes the final mask ineffective.

In summary, BlendMask and SOLO have the best weed segmentation performance in a complex field environment. In the case of data enhancement, the *F*_1_ value of BlendMask is 0.47% higher than that of SOLO, and the AP50 value is 0.69% higher. To further explore the ability of these two models to obtain weed phenotypic information, BlendMask and SOLO were replaced by two backbone networks (ResNet50 and ResNet101), and the calculation times under different backbone networks were compared. The results are shown in Table 3.

Table 3 lists the prediction durations of the model for a single picture. When two different backbone networks are selected, the prediction time of BlendMask is 13.4 ms and 13.9 ms lower than that of SOLO, respectively. SOLO is affected by anchor-based methods; similar to FCIS, it distinguishes location information. The BlendMask model uses the fusion method of FCIS and YOLACT and proposes the Blender module, which has a faster processing speed. Therefore, considering the segmentation performance and prediction time, BlendMask shows satisfactory segmentation performance and can achieve fast and accurate weeding.

### 3.2. Optimization of Different Hyperparameters of BlendMask

The parameters of the model greatly affect the performance of the model, so we have changed the following four hyperparameters of BlendMask:

(1) *R*, the resolution of bottom-level RoI;
(2) *M*, the resolution of top-level prediction;
(3) *K*, the number of bases;
(4) The bottom module is composed of the feature of the backbone network or FPN.

Table 4 is a comparison of different resolutions when *K* = 4, and the bottom module is C3 and C5. We set the resolution *R* of the bottom-level RoI to 28 and 56, with an *R/M* ratio from 14 to 4.

It can be seen from Table 4 that when the resolution *R* is 56, AP50 and AP70 are generally higher than 28. Because the bottom level extracts detailed information such as the leaves and plant centres of weeds, the higher the resolution is, the better the clear characteristics of weeds are obtained. Moreover, the increase in resolution has little effect on the prediction speed of the model, so we set *R* to 56. When *R* is 56, AP50 is 0.005 and AP70 is 0.003 higher when the resolution of top-level prediction is 14 than 7, while the prediction time is 2.9 ms higher. The top level extracts the shape and posture of the whole weed and makes a rough prediction. To achieve a better balance between prediction speed and accuracy, we set *R* to 56 and *M* to 7 in the next ablation experiment.

Table 5 lists the comparison of different bases when *R* is set to 56 and *M* is set to 7. We set the number of bases from one to eight, looking for the best performance of the model. From Table 5, we can see that four bases achieve the best performance. In the next ablation experiments, we set the number of bases to 4.

Table 6 lists the feature extraction performance comparison of different bottom modules when *R* is set to 56, *M* is set to seven and *K* is set to four. From Table 6, we can see that using FPN features as input to the bottom module can not only improve the performance of the model but also improve the reasoning speed of the model. FPN is a feature pyramid with good generalization ability and robustness, which is conducive to obtaining high-level semantic features of weeds in unstructured field environments. Since FPN can extract features on larger feature maps, it can obtain details such as weed leaves and plant centres more accurately. In the next experiment, we used a backbone combined with FPN to extract the features of weeds.

### 3.3. Segmentation Results of Weeds with Different Shooting Angles and Leaf Ages

To verify the recognition performance of the model under different leaf ages and different shooting angles, the morphological difference of a plant is large under different shooting angles and different leaf ages. We compared the segmentation results of BlendMask with those of two different backbone networks (ResNet50 and ResNet101) combined with FPN under different leaf ages and shooting angles. We used seven key indexes: precision rate (*P*), recall rate (R), *F*_1_, intersection over the union (*IOU*), average precision (*AP*), mean average precision (*mAP*), and mean intersection over the union (*mIOU*). The test set, which included 600 images, was used to verify the generalization ability of the model; therefore, 600 images without data enhancement were selected for testing. The total test set included 200 images each for the front view, side view, and top view. As shown in Figure 9, the labels a, b, c, a_leaf, b_leaf, c_leaf, and centre were not recognized, and we considered that these labels were identified as the background.

**Figure 10:**
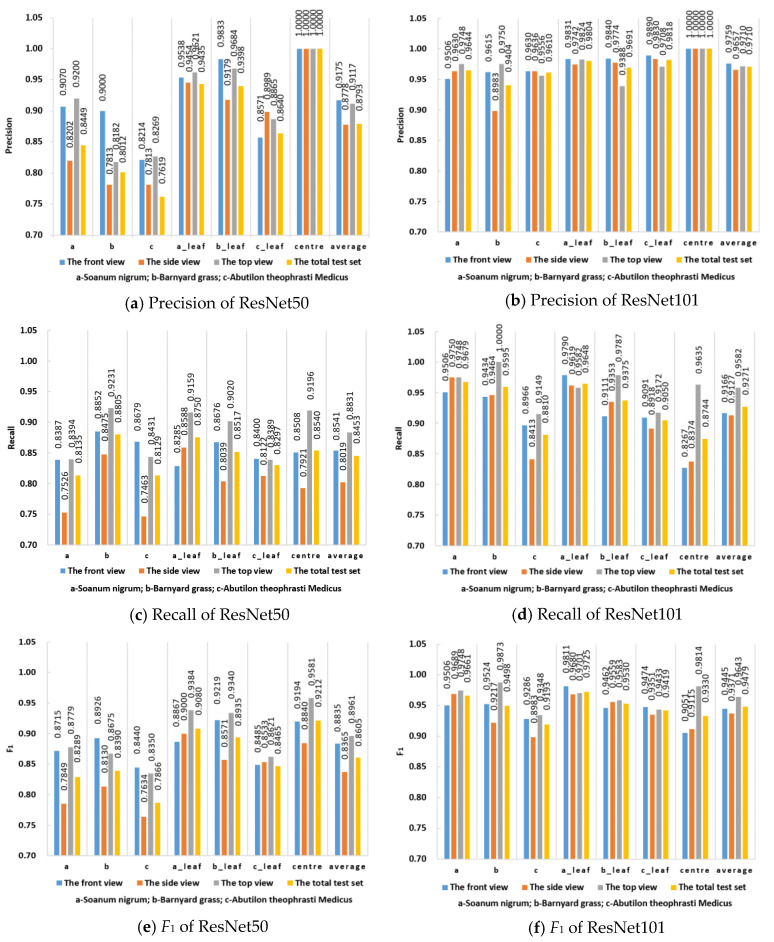
Detection results of BlendMask with pretrained networks in the case of data enhancement. Note: The BlendMask with ResNet50 and ResNet101 frameworks are expressed as ResNet50 and ResNet101, respectively. Centre represents the plant centre of each weed.

Figure 9 shows the confusion matrices of the detection results of the model in the case of data enhancement. This matrix calculates the statistics pertaining to the number of classified images by comparing the actual labels in the validation set data with the predicted types and indicates whether the model can differentiate among different classes. As shown in Figure 9, both ResNet50 and ResNet101 exhibit intuitive common features: The prediction for the leaves of *Solanum nigrum* is highly accurate. *Solanum nigrum* is an annual herb with oval leaves. Moreover, this weed has a large number of leaves, which allows the model to learn more features. Because the model can extract sufficient features from *Solanum nigrum* leaves, *Solanum nigrum* exhibits a high recognition accuracy. However, a part of the leaves of *Solanum nigrum* is predicted to be leaves of *Abutilon theophrasti* Medicus because the leaves of *Abutilon theophrasti* Medicus are also oval, similar to those of *Solanum nigrum*. When collecting data, blurred images may result in insufficient image information extraction. These reasons easily lead to misjudgment of the results, which is not expected because it may lead to errors in leaf age identification. Plant centre recognition accuracy is second only to the leaves of *Solanum nigrum*, and the plant centre is not considered to be another label. Because the plant centre is a special physiological position of weeds, which is obviously different from the characteristics of other categories, there is less misjudgement. According to the confusion matrix, the accuracy of the model, recall and *F*_1_ evaluation index can be further calculated. Figure 10 shows the detection results of the BlendMask model under two backbone networks, three angles, and seven types of labels in the case of data enhancement.

According to Figure 10, the *F*_1_ values of ResNet50 in the front, side, and top views and all the test sets were greater than or equal to 0.7634. In comparison, the *F*_1_ values of ResNet101 were greater than or equal to 0.8983. It can be seen that ResNet101 has higher performance than ResNet50. In ResNet50, the recognition accuracy of barnyard grass leaves was higher than that of *Abutilon theophrasti* Medicus, but in ResNet101, the corresponding accuracies were comparable. Because ResNet50 has fewer convolutional layers than ResNet101, it may not be able to extract sufficient features. ResNet101 increases the depth of the network, so the corresponding *F*_1_ values for barnyard grass (*Echinochloa crus-galli*) and *Abutilon theophrasti* Medicus leaves are high, and the accuracy is comparable. The difference in accuracy between the two types of weed recognition can also be attributed to the shallow network’s inability to extract enough features from the weeds, but the difference between the two types of weed recognition accuracy gradually decreases as the number of layers in the network increases.

In the case of data enhancement, in terms of the *F*_1_ value, the recognition accuracy for the *Solanum nigrum* leaf was the highest. According to the confusion matrix, *Solanum nigrum* leaf have the highest classification accuracy. Analyzed from the subject category of plants, *Solanum nigrum* is an annual herbaceous plant of the Solanaceae family, with upright stems, many branches, oval or heart-shaped leaves, and a large number of leaves. The characteristics of this type of plants are more obvious, which is conducive to the extraction of features by deep learning models.

For the plant centre, the precision values in the front, side, and top views and all the test sets are 1.0000. According to the confusion matrix analysis in Figure 9, since the characteristics of the plant centre are more obvious than those of other categories, the accuracy value is high. The *F*_1_ values of ResNet101 in the front, side, top, and total test sets were 0.9445, 0.9371, 0.9643 and 0.9479, respectively. When ResNet101 was used as the backbone network, the recall values of the front, side and top view test sets were greater than or equal to 0.8267, 0.8374 and 0.9149, respectively. The top view test set exhibited the highest performance in all the classifications.

Since Figure 9 and Figure 10 only indicate the classification performance of the model, the recognition accuracy of the model cannot be determined, and the actual environment in the field is complex, which is expected to influence weed identification. Therefore, the model recognition accuracy is critical to evaluate the model performance. Table 7 presents the detection results for the weeds under different networks and angles with data enhancement.

The *mAP* is a commonly used index in target detection. Table 4 shows that the *mAP* and *mIOU* values of ResNet101 are higher than those of ResNet50, which has good target detection performance, can be applied to the segmentation of small target objects, and can meet the needs of weed segmentation. Therefore, this study selected ResNet101 combined with the FPN framework to extract weed characteristics. For the total test set, when ResNet101 is used as the backbone network, the value of AP50 is 12.8% higher than that of AP70, indicating that a threshold greater than or equal to 0.5 has good detection performance. Using ResNet101 as the backbone network, the AP50 value of the top view is 5.2% and 13.9% higher than that of the front view and side view, respectively. The top view has achieved good detection performance. The *mIOU* is a valuable index to evaluate the segmentation results [43] and is commonly used to evaluate the segmentation performance of the BlendMask model. As shown in Table 7, using ResNet101 as the backbone network, the *mIOU* values of the top view are 4.5% and 5.9% higher than those of the front view and side view, respectively, and good segmentation results are still achieved.

**Figure 11:**
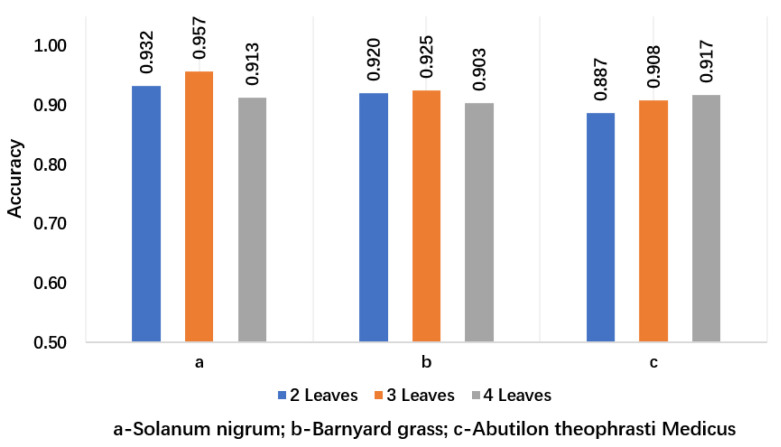
Accuracy for different leaf ages of BlendMask in the case of data enhancement.

**Figure 12:**
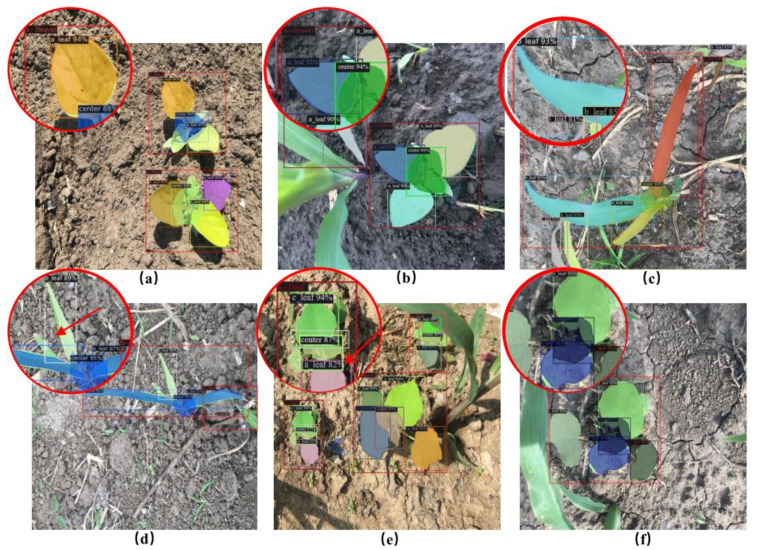
Segmentation results of three weeds. Note: (**a**,**b**) for the identification effect of Solanum nigrum, (**c**,**d**) for the identification effect of barnyard grass, (**e**,**f**) for the identification effect of Abutilon theophrasti Medicus.The recognition accuracy of barnyard grass (*Echinochloa crus-galli*) at the four leaves stage was 0.017 and 0.022 lower than that at two leaves and three leaves, respectively. As shown in Figure 12c,d, when barnyard grass (*Echinochloa crus-galli*) had four leaves, the bottom of the main stem would mostly have some small leaves, which were small and easily concealed by the upper leaves, bringing great difficulties to leaf age recognition. Although the BlendMask model has great advantages in solving complex field environment problems, the effect is still not ideal for this situation, which easily leads to misjudgment of leaf age. The 2-leaf age recognition accuracy rate of *Abutilon theophrasti* Medicus was the lowest among all categories, with a value of 0.887. As shown in Figure 12e, the leaves of *Abutilon theophrasti* Medicus are elliptical, making them similar to the leaves of *Solanum nigrum*, and causing some leaves to be misidentified. The recognition accuracy of *Abutilon theophrasti* Medicus was higher than that of the other two ages on four leaves. As shown in Figure 12f, there were more leaves on four leaves, the petiole of *Abutilon theophrasti* Medicus was longer, and the lower leaves were not easily blocked.

Among the three orthogonal angles, more comprehensive weed phenotype information can be obtained from the perspective of the top view; specifically, the plant centre of the weeds can be identified more clearly. In contrast, the side and front views cannot clearly observe the plant centre. Consequently, the detection accuracy for the top view angle is higher than that for the other angles. Nevertheless, when intelligent agricultural equipment is employed in the field, the camera is usually fixed at an angle, although the position and shape of the weeds in the field are complex and changeable. When the machine is moving, the imaging angle of the weeds changes, and the imaging angle differs owing to the different positions of the weeds. The information of the side and front views is exposed at certain angles; therefore, obtaining the images of the side and front views can help the model accurately segment the weeds. Constructing datasets from different perspectives can enable the model to adapt to the job requirements of different scenarios. To verify the accuracy of this method for leaf age identification, Figure 11 shows the accuracy of different leaf age identification in the case of data enhancement and ResNet101 combined with FPN. Figure 12 lists some of the results of leaf age identification for the three weeds.

To obtain the accuracy of leaf age identification, we used a test set containing 900 unenhanced images, including 300 for *Solanum nigrum*, 300 for barnyard grass (*Echinochloa crus-galli*) and 300 for *Abutilon theophrasti* Medicus. In the *Solanum nigrum* data set, there were 100 weeds with two leaves, three leaves and four leaves, and the remaining two data sets were also set in the same way. The accuracy of leaf identification was determined by comparing the leaf value calculated by the computer with the leaf value on the label when the data were collected.

From Figure 11, we can see that the accuracy of leaf identification of the three weeds was higher than 88%. From the perspective of the growth stage of weeds, the average recognition accuracy of these three weeds at 2 leaf age was 0.913, the average recognition accuracy at 3 leaf age was 0.930, and the average recognition accuracy at 4 leaf age was 0.911. In particular, the recognition accuracy of three leaves of *Solanum nigrum* was 0.957, which is the highest among all leaves. Figure 12a,b show that *Solanum nigrum* leaves are mostly grown from the same centre, and fewer leaves are occluded below.

At present, the treatment of weeds in the field mostly involves weed classification and detection [7,8]. However, weed classification can determine only the species of weeds, and the specific position coordinates of the weeds cannot be obtained; thus, the exact target cannot be sprayed. Weed detection can facilitate the drawing of the bounding box of weeds [12]. However, weeds exhibit an irregular shape and size, which may cause the machine to be inaccurate with respect to the target, resulting in certain herbicides falling to the ground and not being absorbed by the weeds; this aspect may lead to environmental pollution and wastage of the herbicide. As a kind of deep learning model, instance segmentation can detect the target pixel by pixel, thereby solving the problems of blade adhesion and occlusion. Moreover, the leaf age of weeds and the position of the plant centre can be obtained accurately. The data for this study were obtained from a complex field environment, while Bell and Dobrescu et al. carried out a lot of studies on plant leaf counts [15,16,17], but they were all taken in an indoor environment where the backgrounds were often pure and the illumination is uniform. Studying the field environment can help make the model more suitable for practical applications. Wang and Huang et al. identified the central region of maize and rice, respectively, which corresponded to the protected area for mechanical weeding and which was also the centre of the plant [44,45]. It is worth noting that the morphological and structural characteristics of maize and rice are relatively uniform, so the characteristics of the central area are more obvious. However, weeds are polymorphic, and the morphology of weeds of different varieties and leaf ages is quite different, and the growth position is random and variable.

Only a few of the existing studies on plant phenotypes are specific to weed phenotypes. However, weeds of different leaf ages require different doses of herbicides; therefore, it is of significance to obtain information on weed leaf ages to reduce the amount of herbicides. In the Northeast Plain of China, the main economic crops are maize, soybeans, and wheat, which are susceptible to annual and perennial weeds. Controlling annual and perennial weeds can increase crop yields and reduce the likelihood of damage caused by weeds in the second year [46]. Moreover, studying the interaction between the plant phenotype and vision through effective phenotypic analysis can help provide information regarding plant growth and morphological changes.

The employed DCNN model was used to segment only three kinds of weeds, but there are still differences in detail between different kinds of weeds, so it is necessary to expand the kinds of weeds, collect and segment the images of field crops, and increase the number of datasets. The model can achieve a higher segmentation accuracy. Moreover, the obtained leaf age of economic crops can provide a basis for crop fertilization. For certain plants, the plant centre is the pollination area of flowers, and segmentation of this part can provide valuable guidance for subsequent studies. BlendMask failed to segment weeds close to the edge of the image in the weed test image, but continuous video input can help eliminate the edge effects in field applications.

## 4. Conclusions

(1) In this paper, we segmented weeds by the optimized BlendMask model to obtain the weed species, leaf age and plant centre. The *mIOU* value of the method can reach 0.607, the *F*_1_ value of the plant centre is 0.9330, and the recognition accuracy of leaf age can reach 0.957.
(2) Through the comparison of different instance segmentation models and the optimization of hyperparameters, the AP50 value of the BlendMask model can reach 0.720, the single sample time is 114.6 ms, and the best segmentation result is obtained. Data enhancement can increase the AP50 value of the model by 2.47%, and the *mIOU* value of the model can be increased by 10.5% by using ResNet101 combined with FPN architecture for feature extraction. It is proven that the comprehensive application of model comparison, parameter optimization, data enhancement, and replacement of the underlying network are effective methods for solving the identification of leaf age and central area in complex environments. This method can provide a reference for obtaining phenotypic information in similar complex field environments.
(3) The top view obtains the best segmentation performance, and the *mIOU* values of the top view were 4.5% and 5.9% higher than those of the front view and the side view, respectively. Due to serious occlusion, the recognition accuracy of barnyard grass (*Echinochloa crus-galli*) on four leaves was 1.7% and 2.2% lower than that on two leaves and three leaves, respectively. Leaf morphological structure will affect leaf age. The leaf age recognition accuracy of *Solanum nigrum* is 0.957, which is the highest in all categories. This result indicated that the shooting angle, growth distribution characteristics and leaf morphological structure of weeds had an important influence on the recognition performance, which provided ideas and references for subsequent related research and model improvement.

Therefore, the weed information obtained in this study is of great significance for precision variable weeding, and the dataset and research results may provide important resources for future plant phenotype research. Phenotypic information such as weed species, leaf age and central region determine the vital activity of weeds and also influence the competitive relationship between weeds and between weeds and crops, which is important for the development of precise weed control strategies. Future research will be focused on evaluating image datasets that cover a wider range of weeds and crop varieties, explore the competitive relationship between phenotypic parameters of weeds and crops, improving the efficiency of the model, and deploying the trained model on the mobile platform of the spray system for weeding. The proposed study combines artificial intelligence technology with agronomic research concepts, and the findings can facilitate the development of intelligent agriculture.

## Figures and Tables

**Table 1:** List of images containing the environmental information for the experiment.

Date	Images	Tmax (°C)	Tmin (°C)	Weather	Front View Images	Top View Images	Side View Images
25 May 2020	498	30	17	Cloud	198	160	140
30 May 2020	530	22	11	Cloud	192	186	152
31 May 2020	441	21	15	Sunny	148	140	153
4 June 2020	452	24	16	Cloud	142	162	148
8 June 2020	456	24	18	Rain	139	169	148
10 June 2020	557	25	16	Cloud	213	190	154
14 June 2020	462	24	14	Rain	146	171	145
17 June 2020	454	25	13	Cloud	152	154	148
21 June 2020	489	26	14	Cloud	182	165	142
23 June 2020	460	25	15	Sunny	145	157	158
26 June 2020	522	26	17	Cloud	159	176	187
29 June 2020	535	23	15	Cloud	152	214	169
Total	5856	\	\	\	1968	2044	1844

**Table 2:** Characteristic parameters of the pretraining network.

Network	Depth	Size (MB)	Parameters (millions)	Image Input Size	Feature Extraction Layer
ResNet50	50	96	25.6	224 × 224	block_13_expand_relu
ResNet101	101	167	44.6	224 × 224	mixed7

**Table 3:** Prediction time of different models under different backbone networks.

Network	Times (ms)	
	ResNet50	ResNet101
SOLO	102.7	128.5
BlendMask	89.3	114.6

**Table 4:** Comparison of different resolutions.

R	M	AP50	AP70	Time (ms)
28	2	0.652	0.505	85.7
	4	0.664	0.517	86.1
	7	0.677	0.521	88.3
56	4	0.685	0.532	86.3
	7	0.693	0.535	88.2
	14	0.698	0.538	91.1

*Notes*: We set the bases (*K*) of the model to 4 and use C3 and C5 for the bottom module from the backbone network ResNet101. By changing the resolution of the bottom-level RoI and the top-level prediction to compare the performance of the model.

**Table 5:** Comparison of different bases.

K	1	2	4	8
AP50	0.645	0.672	0.693	0.663
AP70	0.497	0.504	0.535	0.524

*Note*: We set *R* to 56 and *M* to 7.

**Table 6:** Comparison of the bottom feature locations from the backbone or FPN.

	Feature	*M*	Time (ms)	AP50	AP70
Backbone	C3,C5	7	88.3	0.653	0.507
		14	91.1	0.657	0.519
FPN	P3,P5	7	84.9	0.664	0.523
		14	89.5	0.667	0.523

*Notes*: The resolution of top-level prediction is set to 56×56, the resolution of bottom-level RoI is set to 7 and the number of bases is set to 4.

**Table 7:** Detection results for the weeds under different networks and angles with data enhancement.

Network		ResNet50	ResNet101
Front view	AP50	0.573	0.732
	AP70	0.485	0.602
	*mIOU*	0.482	0.597
Side view	AP50	0.564	0.645
	AP70	0.472	0.540
	*mIOU*	0.472	0.583
Top view	AP50	0.637	0.784
	AP70	0.521	0.633
	*mIOU*	0.553	0.642
Total test set	AP50	0.591	0.720
	AP70	0.493	0.592
	*mIOU*	0.502	0.607

## Data Availability

Given that the data used in this study were self-collected, the dataset is being further improved. Thus, the dataset is unavailable at present.

## Nomenclature

**Symbols**	
*AP*	Average precision
AP50	*mAP* with the IOU threshold of 0.5
AP70	*mAP* with the IOU threshold of 0.7
CNN	Convolutional neural network
DCNN	Deep convolutional neural network
*F*_1_	Metric function to balance *P* and *R*
Faster R-CNN	Faster regions with convolutional neural network features
FC	Fully convolutional
FCIS	Fully convolutional instance-aware semantic segmentation
FCN	Fully convolutional network
FCOS	Fully convolutional one-stage object detection
*FN*	False negative
*FP*	False positive
FPN	Feature pyramid network
GPU	Graphic processing unit
*IOU*	Intersection over the union
*mAP*	Mean average precision
Mask R-CNN	Mask regions with convolutional neural network features
*mIOU*	Mean intersection over the union
*P*	Precision rate
*R*	Recall rate
ROI	Region of interest
RPN	Region proposal network
Tmax	Maximum temperature
Tmin	Minimum temperature
*TN*	True negative
*TP*	True positive

## References

[1] Slaughter D.C., Giles D.K., Downey D. (2008). Autonomous robotic weed control systems: A review. Comput. Electron. Agric..

[2] Bakhshipour A., Jafari A., Nassiri S.M., Zare D. (2017). Weed segmentation using texture features extracted from wavelet sub-images. Biosyst. Eng..

[3] Taiz L., Zeiger E., Møller I.M., Murphy A. (2015). Plant Physiology and Development.

[4] Xiu L., Yu-quan D., Jing-bo L., Yun-yun Z., Chen-zhong J. (2017). Sensitivity of Barnyard Grass at Different Leaf Stage to Bispyribac-Sodium and Cyhalofop-Butyl. J. Weeds.

[5] Zeisler-Diehl V., Muller Y., Schreiber L. (2018). Epicuticular wax on leaf cuticles does not establish the transpiration barrier, which is essentially formed by intracuticular wax. J. Plant Physiol..

[6] Garcia-Santillan I.D., Pajares G. (2018). On-line crop/weed discrimination through the Mahalanobis distance from images in maize fields. Biosyst. Eng..

[7] Jeon H.Y., Tian L.F., Zhu H.P. (2011). Robust Crop and Weed Segmentation under Uncontrolled Outdoor Illumination. Sensors.

[8] Bossu J., Gee C., Jones G., Truchetet F. (2009). Wavelet transform to discriminate between crop and weed in perspective agronomic images. Comput. Electron. Agric..

[9] Eddy P.R., Smith A.M., Hill B.D., Peddle D.R., Blackshaw R.E. (2014). Weed and crop discrimination using hyperspectral image data and reduced bandsets. Can. J. Remote Sens..

[10] Bakhshipour A., Jafari A. (2018). Evaluation of support vector machine and artificial neural networks in weed detection using shape features. Comput. Electron. Agric..

[11] Chen Y., Wu Z., Zhao B., Fan C., Shi S. (2021). Weed and Corn Seedling Detection in Field Based on Multi Feature Fusion and Support Vector Machine. Sensors.

[12] Quan L.Z., Feng H.Q., Li Y.J., Wang Q., Zhang C.B., Liu J.G., Yuan Z.Y. (2019). Maize seedling detection under different growth stages and complex field environments based on an improved Faster R-CNN. Biosyst. Eng..

[13] Bah M.D., Hafiane A., Canals R. (2018). Deep Learning with Unsupervised Data Labeling for Weed Detection in Line Crops in UAV Images. Remote Sens..

[14] Li Z., Guo R., Li M., Chen Y., Li G. (2020). A review of computer vision technologies for plant phenotyping. Comput. Electron. Agric..

[15] Bell J., Dee H.M. (2019). Leaf segmentation through the classification of edges. arXiv.

[16] Dobrescu A., Giuffrida M.V., Tsaftaris S.A. (2020). Doing More With Less: A Multitask Deep Learning Approach in Plant Phenotyping. Front. Plant Sci..

[17] Ubbens J., Cieslak M., Prusinkiewicz P., Stavness I. (2018). The use of plant models in deep learning: An application to leaf counting in rosette plants. Plant Methods.

[18] Shelhamer E., Long J., Darrell T. (2017). Fully Convolutional Networks for Semantic Segmentation. IEEE Trans. Pattern Anal. Mach. Intell..

[19] Santos T.T., Souza L.L.D., Santos A.A.D., Avila S. (2020). Grape detection, segmentation, and tracking using deep neural networks and three-dimensional association. Comput. Electron. Agric..

[20] Buayai P., Saikaew K.R., Mao X. (2021). End-to-End Automatic Berry Counting for Table Grape Thinning. IEEE Access.

[21] Gené-Mola J., Sanz R., Rosell-Polo J.R., Rubió J.R.M., Lopez E.G. (2020). Fruit detection and 3D location using instance segmentation neural networks and structure-from-motion photogrammetry. Comput. Electron. Agric..

[22] He K.M., Gkioxari G., Dollar P., Girshick R. (2020). Mask R-CNN. IEEE Trans. Pattern Anal. Mach. Intell..

[23] Tian Y.N., Yang G.D., Wang Z., Li E., Liang Z.Z. (2020). Instance segmentation of apple flowers using the improved mask R-CNN model. Biosyst. Eng..

[24] Yu Y., Zhang K.L., Yang L., Zhang D.X. (2019). Fruit detection for strawberry harvesting robot in non-structural environment based on Mask-RCNN. Comput. Electron. Agric..

[25] Lin T.-Y., Maire M., Belongie S., Hays J., Perona P., Ramanan D., Dollár P., Zitnick C.L. (2014). Microsoft coco: Common objects in context. Lecture Notes in Computer Science.

[26] Chen H., Sun K., Tian Z., Shen C., Huang Y., Yan Y. (2020). BlendMask: Top-Down Meets Bottom-Up for Instance Segmentation. arXiv.

[27] Tian Z., Shen C., Chen H., He T. (2019). FCOS: Fully Convolutional One-Stage Object Detection. arXiv.

[28] Mccool C.S., Perez T., Upcroft B. (2017). Mixtures of Lightweight Deep Convolutional Neural Networks: Applied to agricultural robotics. IEEE Robot. Autom. Lett..

[29] Ho D., Tong M., Ienco D., Gaetano R., Maurel P. (2017). Deep Recurrent Neural Networks for mapping winter vegetation quality coverage via multi-temporal SAR Sentinel-1. IEEE Geosci. Remote Sens. Lett..

[30] Gonzalez S., Arellano C., Tapia J. (2019). DeepBlueBerry: Quantification of Blueberries in the Wild Using Instance Segmentation. IEEE Access.

[31] Wu D., Lv S., Jiang M., Song H. (2020). Using channel pruning-based YOLO v4 deep learning algorithm for the real-time and accurate detection of apple flowers in natural environments. Comput. Electron. Agric..

[32] Ienco D., Gaetano R., Dupaquier C., Maurel P. (2017). Land Cover Classification via Multitemporal Spatial Data by Deep Recurrent Neural Networks. IEEE Geosci. Remote Sens. Lett..

[33] Krizhevsky A., Sutskever I., Hinton G.E. (2017). ImageNet classification with deep convolutional neural networks. Commun. ACM.

[34] Dutta A., Gupta A., Zisserman A. VGG Image Annotator (VIA). https://www.robots.ox.ac.uk/~vgg/software/via/.

[35] Lu J., Behbood V., Hao P., Zuo H., Xue S., Zhang G.Q. (2015). Transfer learning using computational intelligence: A survey. Knowl. Based Syst..

[36] Al-Najjar H.A.H., Kalantar B., Pradhan B., Saeidi V., Halin A.A., Ueda N., Mansor S. (2019). Land Cover Classification from fused DSM and UAV Images Using Convolutional Neural Networks. Remote Sens..

[37] Zhang E., Zhang Y., Liu L., ÖZsu M.T. (2009). Average Precision. Encyclopedia of Database Systems.

[38] Ren S.Q., He K.M., Girshick R., Sun J. (2017). Faster R-CNN: Towards Real-Time Object Detection with Region Proposal Networks. IEEE Trans. Pattern Anal. Mach. Intell..

[39] Wang X., Kong T., Shen C., Jiang Y., Li L. (2019). SOLO: Segmenting Objects by Locations. arXiv.

[40] Xie E., Sun P., Song X., Wang W., Liang D., Shen C., Luo P. (2019). PolarMask: Single Shot Instance Segmentation with Polar Representation. arXiv.

[41] Lee Y., Park J. CenterMask: Real-Time Anchor-Free Instance Segmentation. Proceedings of the 2020 IEEE/CVF Conference on Computer Vision and Pattern Recognition (CVPR).

[42] Bolya D., Zhou C., Xiao F., Lee Y.J. (2019). YOLACT: Real-time Instance Segmentation. arXiv.

[43] Garcia-Garcia A., Orts-Escolano S., Oprea S., Villena-Martinez V., Garcia-Rodriguez J. (2017). A Review on Deep Learning Techniques Applied to Semantic Segmentation. arXiv.

[44] Wang X., Zhang H., Chen Y., Lightfoot D.A. (2018). Research on maize canopy center recognition based on nonsignificant color difference segmentation. PLoS ONE.

[45] Huang S., Wu S., Sun C., Ma X., Qi L. (2020). Deep localization model for intra-row crop detection in paddy field. Comput. Electron. Agric..

[46] Lehoczky E., Nagy P., Lencse T., Toth V., Kismanyoky A. (2009). Investigation of the Damage Caused by Weeds Competing with Maize for Nutrients. Commun. Soil Sci. Plant Anal..

